# Polyphenol extract from evening primrose pomace alleviates experimental colitis after intracolonic and oral administration in mice

**DOI:** 10.1007/s00210-014-1025-x

**Published:** 2014-07-31

**Authors:** M. Sałaga, U. Lewandowska, D. Sosnowska, P. K. Zakrzewski, A. I. Cygankiewicz, A. Piechota-Polańczyk, M. Sobczak, P. Mosinska, Chunqiu Chen, W. M. Krajewska, J. Fichna

**Affiliations:** 1 Department of Biochemistry, Faculty of Medicine, Medical University of Lodz, Mazowiecka 6/8, 92-215 Lodz, Poland; 2 Institute of Technical Biochemistry, Department of Biotechnology and Food Sciences, Technical University of Lodz, Lodz, Poland; 3 Department of Cytobiochemistry, Faculty of Biology and Environmental Protection, University of Lodz, Lodz, Poland; 4 Department of Gastroenterological Surgery, Tenth People’s Hospital of Shanghai, School of Medicine, Tongji University, Shanghai, China

**Keywords:** Evening primrose, Experimental colitis, Inflammatory bowel diseases

## Abstract

*Oenothera paradoxa* (EP) preparations are commonly used in folk medicine to treat skin diseases, neuralgia, and gastrointestinal (GI) disorders. Several reports suggested that EP preparations exhibit potent anti-inflammatory and antioxidant activities both in vitro and in vivo. Here, we aimed to characterize the action of EP pomace polyphenol extract in mouse model of colitis. We analyzed the composition of EP pomace polyphenol extract using reversed phase HPLC system and ultra-performance liquid chromatography (UPLC) system coupled with a quadrupole-time of flight (Q-TOF) MS instrument. Then, we used a well-established animal model of 2,4,6-trinitrobenzenesulfonic acid (TNBS)-induced colitis to determine the anti-inflammatory action of EP pomace polyphenol extract. We also investigated the effect of the EP pomace polyphenol extract on pro-inflammatory (IL-1β and TNF-α) cytokine mRNA levels and hydrogen peroxide concentration in the inflamed colon. Administration of EP pomace polyphenol extract significantly improved macroscopic and microscopic damage scores, as well as myeloperoxidase (MPO) activity in TNBS-treated mice. The anti-inflammatory effect of the extract was observed after intracolonic and oral administration and was dose-dependent. Significant reduction of tissue hydrogen peroxide level after treatment with EP pomace polyphenol extract suggests that its therapeutic effect is a result of free radical scavenging. This novel finding indicates that the application of the EP pomace polyphenol extract in patients with inflammatory bowel diseases (IBDs) may become an attractive supplementary treatment for conventional anti-inflammatory therapy.

## Introduction

Evening primrose (*Oenothera paradoxa*) is a biennial herb originating from Mexico and Central America. Throughout the years, it has become a widespread plant occurring in both Americas, Europe, and parts of Asia (Bayles and Usatine 2009). The Native Americans valued evening primrose stem and leaf juices as topical remedies to alleviate cutaneous inflammation (Bayles and Usatine 2009). An extract made of *O. paradoxa* (EP) roots was applied to treat obesity and abdominal pain (Singh et al. 2012). EP has also been used in folk medicine as a remedy for neuralgia, skin, liver, kidney, and gastrointestinal (GI) diseases (Singh et al. 2012).

Several secondary metabolites, which are present in various parts of the plant, contribute to the therapeutic actions of EP. For instance, anti-inflammatory and radical scavenging triterpenoids and low molecular weight phenolic antioxidants occur in EP seeds, while flavonoids with anticancer activity can be found in the whole plant (Knorr and Hamburger 2004; Montserrat-de la Paz et al. 2012; Singh et al. 2012; Wettasinghe et al. 2002). Clinical evidence for therapeutically relevant action of EP has recently been reported. The EP oil is best known for its beneficial effect on the chronic inflammatory disorders, such as atopic dermatitis and rheumatoid arthritis (Bayles and Usatine 2009). It is also used as an analgesic agent in breast pain (mastalgia) (Bayles and Usatine 2009). Moreover, the potential application of EP oil in diabetes mellitus, asthma, and schizophrenia is currently being investigated (Bayles and Usatine 2009). Several reports suggest that use of various EP preparations exhibits therapeutic properties in the GI tract (al-Shabanah 1997; Gorlach et al. 2011; Greenfield et al. 1993).

Inflammatory bowel diseases (IBDs) are a group of chronic inflammatory GI disorders, consisting of Crohn’s disease (CD) and ulcerative colitis (UC) that may cause a substantial worsening of life quality (Ng et al. 2013). Currently available pharmacotherapeutic treatment strategies for IBD comprise corticosteroids, immunosuppressants, and antibodies against tumor necrosis factor (TNF-α) (Ng et al. 2013).

In the recent years, the use of complementary and alternative medicine (CAM) has been perceived as attractive by patients with IBD and is systematically becoming more and more popular. Population-based and cohort studies have shown that prevalence of current or past CAM use in adult IBD populations from North America and Europe ranges from 21 up to 60 % (Ng et al. 2013).

The aim of the present study was to extend previously reported findings on the therapeutic significance of EP preparations by characterizing the anti-inflammatory potential of polyphenol extract from EP pomace, which is a residue that remains after oil extrusion from the seeds.

## Materials and methods

### Chemicals

Acetonitrile, elagic acid, (+)-catechin, formic acid, gallic acid, methanol, methyl gallate, protocatechuic acid, and vanillin were purchased from Sigma-Aldrich Chemicals Co. (Poznan, Poland) and quercetin glucoside from Extrasynthese (Lyon, France). All other chemicals were reagent-grade products purchased from POCH (Gliwice, Poland).

### Plant materials

Evening primrose (*O. paradoxa*) pomace defatted seeds were obtained from Agropharm S.A. (Tuszyn, Poland). Pomace contains waste material from the cold pressing of evening primrose oil. Such material is almost completely deprived from lipids and fatty acids. Moreover, remaining lipids and fatty acids were removed from the material during extraction, which is described in detail below.

### Preparation of the polyphenol extract

Evening primrose pomace (90 g) obtained in the process of oil pressing was milled and defatted with hexane. The first extraction lasted 30 min and was followed by two 15-min extractions. The ratio of the plant material to hexane was 1:5 (*w*/*v*) in the first extraction and 1:2.5 (*w*/*v*) in the second and third. The hexane extracts were centrifuged (15 min, 4,000 rpm), and defatted seeds were dried at room temperature for 24 h. Polyphenols were extracted from the defatted seeds (81.6 g) with the use of a 70 % aqueous solution of ethanol at room temperature. The ratio of plant material to ethanol solution was 1:10 (*w*/*v*) in the first extraction and 1:5 (*w*/*v*) in the next two extractions. The ethanol extracts were centrifuged (15 min, 4,000 rpm), combined, and evaporated under vacuum at ≤40 °C (Rotavapor RII, BUCHI, Flawil, Switzerland). The obtained aqueous solution of the evening primrose polyphenols was lyophilized (Alpha 1-2 LD plus, Christ, Osterode, Germany). The final dry extract (9.91 g) was stored at 4 °C prior to further analyses.

### Total phenolic content

The total phenolic content of the polyphenol extract was determined using Folin-Ciocalteu reagent, based on the method described by Bordonaba and Terry (2008) with some modification. Accurately weighed 20 mg of extracts was dissolved in 10 mL of 10 % aqueous dimethyl sulfoxide (DMSO); then, 0.1–0.2 mL were mixed with 25 mL of water, 0.5 mL of Folin-Ciocalteu reagent, and 5 mL of 20 % sodium carbonate and made up to 50 mL with distilled water. The mixture was kept for 20 min at room temperature, after which the absorbance was read at 760 nm. (+)-Catechin was used as a reference standard, and the results were expresses as (+)-catechin equivalents/g of dry extract.

### Total flavan-3-ol content

The vanillin assay was performed as described by Swain and Hillis (1959), with some modification. Briefly, a volume of 2 mL of a known dilution of the preparation solution in water was placed in two test tubes, and 4 mL of 1 % (*w*/*v*) vanillin in 70 % sulfuric acid (A) or 4 mL of 70 % sulfuric acid (B) was added to a sample. Additionally, a blank was prepared by mixing 2 mL of water with vanillin solution (C). All test tubes were shaken in a bath of cold water to prevent the temperature from rising above 35 °C. After incubation in cold water for 15 min, the absorbance of samples A, B, and C was read at 500 nm against a mixture of 2 mL of water and 4 mL of 70 % sulfuric acid. The final absorbance is equal to the difference (A − B − C). Flavanol content was calculated from a calibration curve, using (+)-catechin as standard. Results were expressed as milligram catechin equivalents/g of dry extract.

### Hydrolyzable tannin content

Hydrolyzable tannin content in the obtained extract was estimated by an HPLC method after acid hydrolysis of tannins into methyl gallate according to Hartzfeld et al. (2002) with some modifications. Briefly, 5–10-mg samples of dry polyphenol extract were weighed into 25-mL Pyrex screw top tubes with Teflon cap liners, and 4 mL of methanol was added, followed by 0.4 mL of concentrated sulfuric acid. The samples were placed in a heating block previously preheated to 85 °C and were allowed to react for 20 h. After cooling, 0.4 mL of ethanolamine (commercial preparation, 100 % ethanolamine) was added to the mixture, and the volume was adjusted to 10 mL with distilled water. Prior to HPLC analysis, the samples were centrifuged for 3 min (13,000 rpm) and filtered through a 0.45-μm syringe filter Minisart RC4 (Sartorius, Goettingen, Germany). Methyl gallate was determined using analytical reversed phase HPLC system (Waters, Milford, MA, USA) with autosampler 2707 and binary HPLC pump 1525 coupled to a 996 photodiode array detector (2998), controlled by Waters Breeze 2 software. Separation was performed on a SYMMETRY C18 (250 mm × 4.6 mm, 5 μm) column (Waters, Milford, MA, USA). The binary mobile phase according to Dyrby et al. (2001) consisted of water and formic acid in the ratio of 90:10 (*v*/*v*), respectively (solvent A), water, acetonitrile, and formic acid in the ratio of 49:50:1 (*v*/*v*/*v*), respectively (solvent B). The separation of phenolic was performed using the following gradient program with a flow rate of 1 mL/min: 0 min, 88 % A + 12 % B; 26 min, 70 % A + 30 % B; 40–43 min, 0 % A + 100 % B; and 48–50 min, 88 % A + 12 % B. Detection was performed by scanning from 200 to 550 nm. The methyl gallate (detection at 280 nm) was analyzed and quantified as methyl gallate equivalents/g of dry extract.

### UPLC-Q-TOF-MS conditions

Identification of polyphenol compounds was performed on an Acquity ultra-performance liquid chromatography (UPLC) system coupled with a quadrupole-time of flight (Q-TOF) MS instrument (UPLC/Synapt Q-TOF MS, Waters, Milford, MA, USA) with an electrospray ionization (ESI) source according to Wojnicz et al. (2012). Separation was achieved on an AcquityTM BEH C18 column (100 mm × 2.1 mm i.d., 1.7 μm; Waters). Mobile phase was a mixture of 4.5 % formic acid (A) and acetonitrile (B). The gradient program was as follows: initial conditions 99 % (A); 12 min, 75 % (A); 12.5 min, 100 % (B); 13.5 min, 99 % (A). The flow rate was 0.45 mL/min. The major operating parameters for the Q-TOF MS were set as follows: capillary voltage, 2.0 kV; cone voltage, 45 V; cone gas flow, 11 L/h; collision energy, 50 eV; source temperature, 100 °C; desolvation temperature, 250 °C; collision gas, argon; desolvation gas, nitrogen; flow rate, 600 L/h; data acquisition range, *m*/*z* 100–1,000 Da; ionization mode, negative.

### HPLC analysis of phenolic compounds

HPLC analysis was performed using analytical reversed phase HPLC system (Dionex, Sunnyvale, CA, USA) with autosampler EWPS-3000SI and pump LPG-3400A coupled to a photodiode array detector (Ultimate 3000), controlled by Chromeleon v. 6.8 software, according to Kucharska (2012). Separation was performed on an Atlantis T3 (250 mm × 4.6 mm i.d., 5 μm; Waters, Dublin, Ireland). The eluent was 4.5 % formic acid (A) and acetonitrile (B). A gradient solvent system was used: 0–1 min, 5 % (B); 1–6 min, 10 % (B); 6–26 min, 20 % (B); and 26–33 min, 100 % (B). The flow rate was 1 mL/min, and the injection volume was 20 μL. Detector was set at 254 nm for elagic acid and protocatechuic acid; 280 nm for gallic acid, flavan-3-ol derivatives, and hydrolyzable tannin derivatives; and 360 nm for flavonol derivatives. The amounts of flavan-3-ol derivatives were expressed as (+)-catechin equivalents, hydrolyzable tannin derivatives as gallic acid equivalents, and flavonols as quercetin equivalents/g of dry extract.

### Animals

Experimentally naive male C57B1/6 mice were obtained from the Animal House of the University of Lodz, Poland. All animals used in experiments weighed 22–30 g. The animals were housed at a constant temperature (22 °C) and maintained under a 12-h light/dark cycle (lights on at 6:00 a.m.) in sawdust-lined plastic cages with access to chow pellets and tap water ad libitum. All animal protocols were in accordance with the European Communities Council Directive of 24 November 1986 (86/609/EEC) and Polish legislation acts concerning animal experimentation. The experimental protocol was approved by the Local Ethics Committee at the Medical University of Lodz (#670/2013). All efforts were made to minimize animal suffering and to reduce the number of animals used.

### Induction of colitis and evaluation of disease progress parameters

Experimental colitis was induced by intracolonic (i.c.) administration of 2,4,6-trinitrobenzenesulfonic acid (TNBS, 4 mg in 0.1 mL of 30 % ethanol in saline), as described earlier by Fichna et al. (2012). Three days after TNBS infusion, animals were sacrificed by cervical dislocation. Then, the colon was isolated, opened longitudinally, rinsed with phosphate-buffered saline (PBS), and immediately examined. Macroscopic colon damage was assessed by an established semiquantitative scoring system by adding individual scores for ulcer, colon shortening, wall thickness, and presence of hemorrhage, fecal blood, and diarrhea, as described before (Fichna et al. 2012). For scoring ulcer, colon shortening, and colon wall thickness, the following scale was used: ulcer, 0.5 points for each 0.5 cm; shortening of the colon, 1 point for >15 %, 2 points for >25 % (based on a mean length of the colon in untreated mice of 7.97 ± 0.21 cm, *n* = 6); the wall thickness was measured in mm, a thickness of *n* mm corresponds to *n* scoring points. The presence of hemorrhage, fecal blood, or diarrhea increased the score by 1 point for each additional feature. Body weight of animals was recorded once daily during whole experiment. All colon samples for further experiments were collected during macroscopic evaluation process.

### Determination of myeloperoxidase activity

Myeloperoxidase (MPO) activity was assessed in the mouse colon specimens according to the method described earlier by Fichna et al. (2012). Briefly, 1-cm segments of the colon were weighed and homogenized in hexadecyltrimethylammonium bromide (HTAB) buffer (0.5 % HTAB in 50-mM potassium phosphate buffer, pH 6.0; 1:20 *w*/*v*) immediately after isolation. The homogenate was centrifuged (15 min, 13,200 *g*, 4 °C). On a 96-well plate, 200 μL of 50-mM potassium phosphate buffer (pH 6.0), containing 0.167 mg/mL of *O*-dianisidine hydrochloride and 0.05 μL of 1 % hydrogen peroxide, was added to 7 μL of supernatant. Absorbance was measured at 450 nm (iMARK Microplate Reader, Biorad, Hertfordshire, UK). All measurements were performed in triplicate. MPO was expressed in milliunits per gram of wet tissue, 1 unit being the quantity of enzyme able to convert 1 μmol of hydrogen peroxide to water in 1 min at room temperature. Units of MPO activity per 1 min were calculated from a standard curve using purified peroxidase enzyme.

### Histology

After the macroscopic scoring, segments of the distal colon were stapled flat, mucosal side up, onto cardboard and fixed in 10 % neutral-buffered formalin for 24 h at 4 °C. Samples were then dehydrated, embedded in paraffin, sectioned at 5 μm, and mounted onto slides. Subsequently, sections were stained with hematoxylin and eosin and examined using Motic AE31 microscope (Ted Pella, Vendelsö, Sweden). Photographs were taken using a digital imaging system consisting of a digital camera (Moticam 2300, Ted Pella, Vendelsö, Sweden) and image analysis software (Motic Images Plus 2.0, Wetzlar, Germany).

A microscopic total damage score was determined based on the presence (score = 1) or absence (score = 0) of goblet cell depletion, the presence (score = 1) or absence (score = 0) of crypt abscesses, the destruction of mucosal architecture (normal = 1, moderate = 2, extensive = 3), the extent of muscle thickening (normal = 1, moderate = 2, extensive = 3), and the presence and degree of cellular infiltration (normal = 1, moderate = 2, transmural = 3).

### Determination of IL-1β and TNF-α mRNA levels

RNA was isolated according to manufacturer’s protocol using PureLink RNA Mini kit (Life Technologies, Carlsbad, CA, USA). Briefly, tissue samples were homogenized in lysis buffer (600 μL), complemented with 1 % 2-mercaptoethanol (St. Louis, MO, USA). Subsequently, the homogenates were centrifuged to clear the solution. Supernatants were placed onto ion exchange columns, and finally, purified total RNA was eluted using diethyl pyrocarbonate (DEPC)-treated water (50 μL). To measure the purity and quantity of isolated RNA, dedicated spectrophotometer (BioPhotometer; Eppendorf, Germany) was used. Total RNA (1 μg) was transcribed onto cDNA with First-Strand cDNA synthesis kit (Fermentas, Burlington, Canada). Quantitative analysis was performed using fluorescently labeled TaqMan probe Mm00434228_m1 for mouse IL-1β, Mm00443260_g1 for mouse TNF-α, and Mm01545399_m1 for mouse hypoxanthine-guanine phosphoribosyltransferase (HPRT) as endogenous control (Life Technologies, Carlsbad, CA, USA) on Mastercycler S realplex 4 apparatus (Eppendorf, Hamburg, Germany) and TaqMan Gene Expression Master Mix (Life Technologies, Carlsbad, CA, USA) in accordance with manufacturer’s protocol. All experiments were performed in triplicate.

The Ct (threshold cycle) values for studied genes were normalized to Ct values obtained for a housekeeping gene HPRT. Relative amount of mRNA copies was calculated using the following equation: 2^−ΔCt^ × 1,000.

### Determination of H_2_O_2_

Briefly, 50 mg of colon tissue fragment was homogenized with 2 mL of 1.15 % potassium chloride. Then, 10-μL aliquot of tissue homogenate was mixed with 90 μL of PBS (pH 7.0) and 100 μL of horseradish peroxidase (1 U/mL) containing 400 μmol homovanilic acid (HRP + HVA assay) or with 90 μL of PBS and 100 μL of 1 U/mL horseradish peroxidase only (HRP assay). Both homogenates were incubated for 60 min at 37 °C. Subsequently, 300 μL of PBS and 125 μL of 0.1 M glycine-NaOH buffer (pH 12.0) with 25 mM EDTA were added to each homogenate sample. Excitation was set at 312 nm, and emission was measured at 420 nm (Perkin Elmer Luminescence Spectrometer, Beaconsfield UK). Readings were converted into H_2_O_2_ concentration using the regression equation: *Y* = 0.012*X*–0.007, where *Y* = H_2_O_2_ concentration in homogenate (μM); *X* = intensity of light emission at 420 nm for HRP + HVA assay reduced by HRP assay emission (arbitrary units, AU). The regression equation was prepared from three series of calibration experiments with ten increasing H_2_O_2_ concentrations (range 10–1,000 μM). The lowest H_2_O_2_ detection was 0.1 nM, with intra-assay variability not exceeding 2 %.

### Pharmacological treatments

The EP pomace polyphenol extract was administered twice daily at the dose of 1, 5, and 10 mg/kg (orally, p.o., or i.c.) in TNBS model, with the first treatment 30 min before the induction of colitis. 5-Aminosalicylic acid (5-ASA), which was used as a positive control for the effect of the EP pomace polyphenol extract in TNBS-induced colitis, was administered at the dose of 5 mg/kg (i.c.) 10 min before TNBS infusion. Saline was used as vehicle and did not influence the observed parameters when given alone. Control animals received vehicle alone (100 μL i.c. or 150 μL p.o.).

### Statistics

Statistical analysis was performed using Prism 5.0 (GraphPad Software Inc., La Jolla, CA, USA). The data are expressed as means ± SEM. Student’s *t* test or one-way ANOVA followed by Newman-Keuls post hoc test were used for analysis. *P* values <0.05 were considered statistically significant.

## Results

### Chemical characteristic of EP pomace polyphenol extract

The polyphenol extract obtained from EP defatted seeds contained hydrolyzable and condensed tannins (Table 1). Hydrolyzable tannins were determined as methyl gallate formed during acid hydrolysis of tannins in methanol. These tannins constituted 6.2 % of the dry extract and 10.14 % of the total polyphenols determined with Folin-Ciocalteu method. Condensed tannin content, determined with vanilin reagent, was twice higher than the content of the hydrolyzable tannins. They accounted for 12.5 % of the dry extract and 20.51 % of the total polyphenols.

**Table 1:** General characteristic of the evening primrose polyphenol extract

	Content (mg/g dry extracts)
Total polyphenols^a^	611.55 ± 33.05
Total flavan-3-ols^b^	125.44 ± 2.15
Gallotannins^c^	62.04 ± 1.17

Values are expressed as mean ± SD, *n* ≥ 3

^a^Determined by Folin-Ciocalteu reagent as (+)-catechin equivalents

^b^Determined by vanillin reagent as (+)-catechin equivalents

^c^Determined by HPLC at 280 nm as methyl gallate

### The UPLC-Q-TOF-MS analysis of EP pomace polyphenol extract

The UPLC-Q-TOF-MS analysis of the extract showed the presence of polyphenol compounds belonging to different groups: flavan-3-ol derivatives (eight compounds, 25.48 mg/g of extract), hydrolyzable tannin derivatives (seven compounds, 38.31 mg/g), phenolic acids (three compounds, 32.45 mg/g), and flavonols (two compounds, 0.51 mg/g)—Table 2. The analysis showed that the predominant compounds in this extract were pentagalloyl glucose, ellagic acid, catechin, and gallic acid, with a lot of accompanying polyphenols in lower concentrations.

**Table 2:** The content (mg/g) and characterization of the phenolic compounds in the extract of EP pomace

Peak number	*t*_R_ (min)	[M-H]^−^ (*m/z*)	Compound	Chemical entity	Quantity (mg/g of dry extract)
1	1.03	169.0163	gallic acid	phenolic acid	9.001
2	1.55	483.0756	digalloyl glucose	hydrolyzable tannins	0.585
3	1.84	153.0204	protocatechuic acid	phenolic acid	4.278
4	2.56	577.1349	procyanidin dimer	flavan-3-ols	1.955
5	2.68	577.1349	procyanidin dimer	flavan-3-ols	0.893
6	2.84	865.1905	procyanidin trimer	flavan-3-ols	6.803
7	2.99	865.1906	procyanidin trimer	flavan-3-ols	
8	3.18	289.0723	catechin	flavan-3-ols	12.255
9	4.14	729.1459	procyanidin dimer gallate	flavan-3-ols	1.596
10	4.81	729.1459	procyanidin dimer gallate	flavan-3-ols	0.484
11	5.01	787.0952	tetragalloyl glucose	hydrolyzable tannins	0.304
12	5.32	441.0835	catechin gallate	flavan-3-ols	1.493
13	5.51	787.0952	tetragalloyl glucose	hydrolyzable tannins	1.347
14	6.25	787.0952	tetragalloyl glucose	hydrolyzable tannins	2.639
15	6.44	787.0952	tetragalloyl glucose	hydrolyzable tannins	3.919
16	6.53	787.0952	tetragalloyl glucose	hydrolyzable tannins	1.571
17	6.63	300.9963	ellagic acid	phenolic acid	19.169
18	7.28	477.0620	quercetin glucuronide	flavonols	0.023
19	7.75	939.0977	pentagalloyl glucose	hydrolyzable tannins	27.945
20	8.18	433.0735	quercetin pentoside	flavonols	0.484

Flavan-3-ol derivatives were determined by HPLC at 280 nm as (+)-catechin, hydrolyzable tannin derivatives were determined at 280 nm as gallic acid, and flavonol derivatives were determined at 360 nm as quercetin glucoside

*s* retention time

### EP pomace polyphenol extract has anti-inflammatory effect in TNBS-induced colitis in mice

**Fig. 1:**
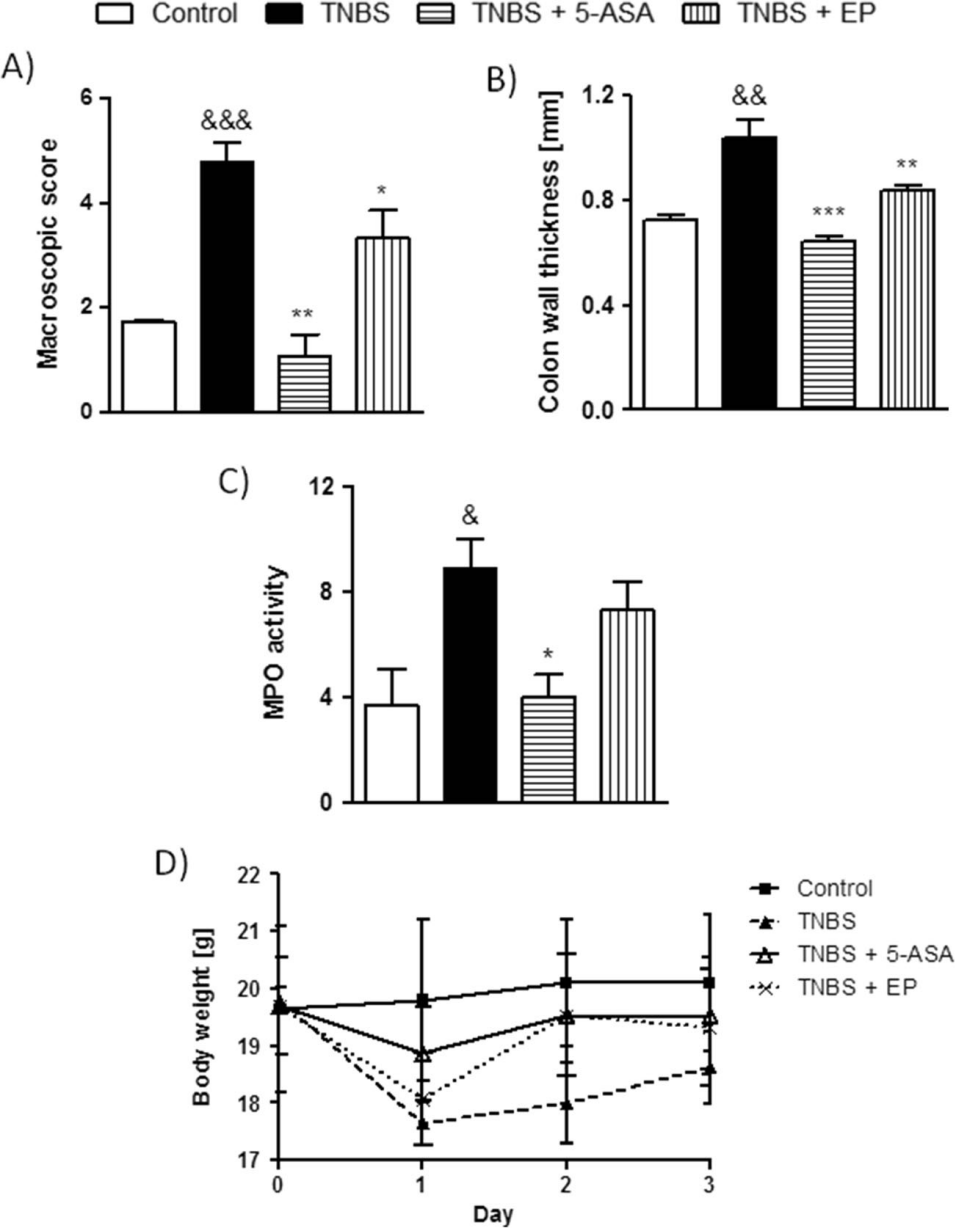
The EP pomace polyphenol extract and 5-ASA (both at the dose of 5 mg/kg, twice daily over 3 days) attenuated TNBS-induced colitis in mice after i.c. administration. *Figure* shows data for macroscopic score (**a**), colon wall thickness (**b**), MPO activity (**c**), and changes in body weight (**d**). &*P* < 0.05, &&*P* < 0.01, &&&*P* < 0.001, as compared to control mice. *P < 0.05, **P < 0.01, ***P < 0.001 versus TNBS-treated animals. Data represent mean ± SEM of 6–8 mice per group

**Fig. 2:**
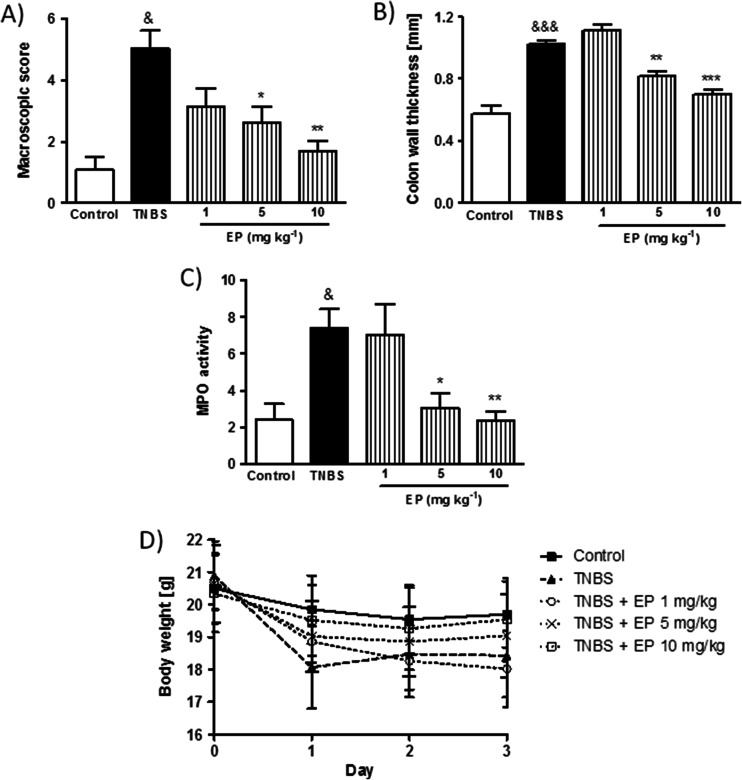
The EP pomace polyphenol extract attenuates colitis in a dose-dependent manner after p.o. administration. *Figure* shows data for macroscopic scores (**a**), MPO activity (**b**), colon wall thickness (**c**), and changes in body weight (**d**). &*P* < 0.05, &&&*P* < 0.001, as compared to control mice. *P < 0.05, **P < 0.01, ***P < 0.001 versus TNBS-treated animals. Data represent mean ± SEM of 6–8 mice per group

Macroscopic damage, colon wall thickness, and MPO activity were assessed 3 days after TNBS treatment and were significantly increased compared with control animals, which received vehicle alone (Fig. 1a–c). 5-ASA, which was used as a reference drug, administered at the dose of 5 mg/kg i.c. (twice daily) significantly attenuated colitis as shown by decreased macroscopic score, reduced colon wall thickness, and MPO activity (Fig. 1a–c). The i.c. administration of the EP pomace polyphenol extract (5 mg/kg, twice daily) significantly reduced total macroscopic damage score and colon wall thickness but did not affect the MPO activity in the TNBS-treated mice (Fig. 1). Of note, mice treated with EP pomace polyphenol extract regained their body weight faster than the vehicle-treated animals. Oral administration of the EP pomace polyphenol extract (5 and 10 mg/kg, twice daily) significantly reduced macroscopic damage score, MPO activity, and colon wall thickness (Fig. 2a–c). Moreover, the anti-inflammatory activity of the extract was dose-dependent. The EP pomace polyphenol extract had no influence on naive animals when administered either orally or i.c.

**Fig. 3:**
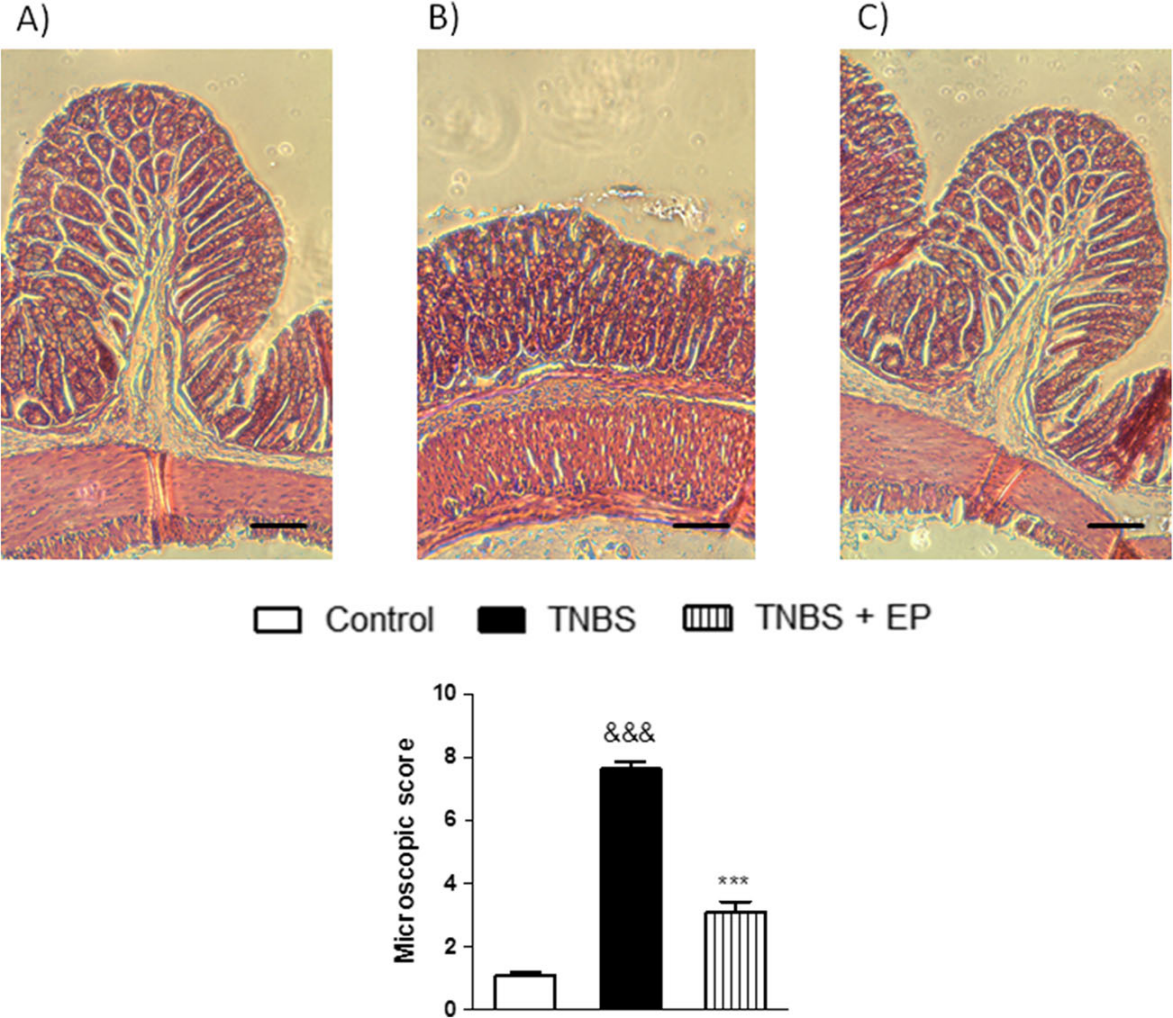
Microscopic total damage score and representative micrographs of hematoxylin and eosin-stained sections of distal colon from **a** control, **b** TNBS, and **c** TNBS + EP (5 mg/kg, twice daily, p.o.)-treated mice. *Scale bar* = 100 μm. &&&*P* < 0.001, as compared with control mice, ***P < 0.001, as compared to TNBS-treated mice. Data represent mean ± SEM of 6–8 mice per group

Microscopic evaluation of colon sections stained with hematoxylin/eosin was in line with observation of the macroscopic parameters (Fig. 3). Histological analysis of sections of distal colon from untreated animals showed intact epithelium, absence of edema, and normal muscle architecture (Fig. 3a). Severe microscopic damage, characterized by loss of mucosal architecture, thickening of smooth muscle, presence of crypt abscesses, and extensive cellular infiltrate, was observed in colon specimens 3-day post-TNBS (Fig. 3b). The histological changes were normalized after treatment with p.o. EP pomace polyphenol extract (Fig. 3c).

### EP pomace polyphenol extract does not affect the level of IL-1β and TNF-α in mouse colon tissue

In order to examine whether the polyphenol extract from EP pomace affects the expression of pro-inflammatory cytokines in intestinal inflammation, we determined the levels of IL-1β and TNF-α mRNA in mouse colon samples. Treatment with TNBS induced a significant elevation of IL-1β mRNA in the colon tissue (Fig. 3a). There was no statistical significance between the level of IL-1β in TNBS-treated animals versus TNBS + EP pomace polyphenol extract at the dose of 5 mg/kg twice daily. However, a tendency toward this reduction may be observed (Fig. 3a). Determination of TNF-α mRNA showed no difference between any of the experimental groups (Fig. 3b).

### EP pomace polyphenol extract reduces the level of hydrogen peroxide in mouse colon tissue

**Fig. 4:**
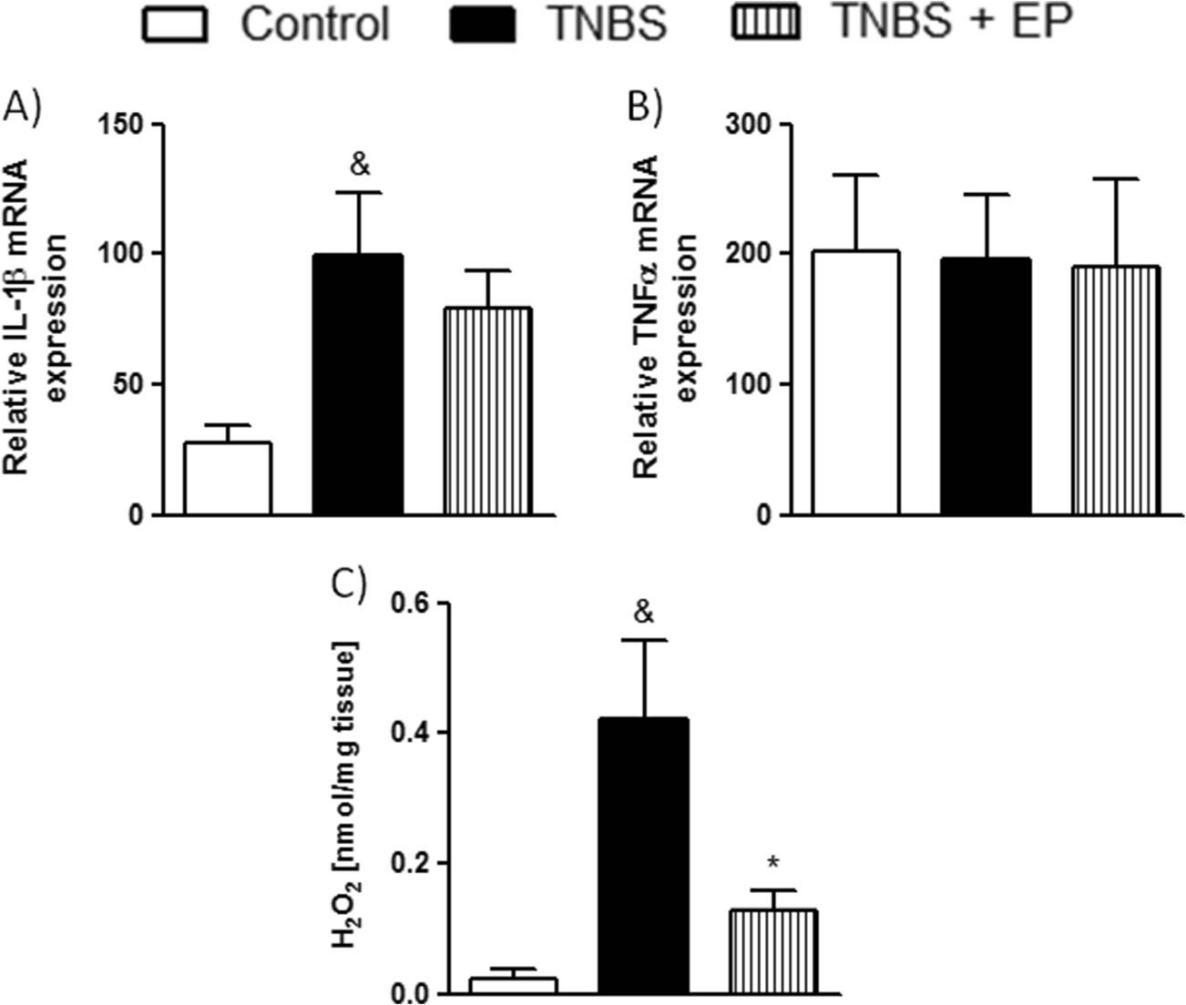
The effect of the EP pomace polyphenol extract (5 mg/kg, twice daily over 3 days, p.o.) on the **a** IL-1β mRNA, **b** TNF-α mRNA, and **c** *Oenothera biennis* H_2_O_2_ levels in colon specimens isolated from TNBS-treated mice. &*P* < 0.05, as compared to control mice. *P < 0.05 versus TNBS-treated animals. Data represent mean ± SEM of 6–8 mice per group

Since we showed that the EP pomace polyphenol extract contains a considerable amount of phenolic antioxidants, the hydrogen peroxide (H_2_O_2_) concentrations were determined in the mouse colon samples as an indicator of the level of oxidative stress (Fig. 4). We observed that the treatment of mice with TNBS substantially raised the levels of H_2_O_2_ suggesting the induction of oxidative stress pathways in the colon tissues (Fig. 3c). Oral administration of EP pomace polyphenol extract for 3 consecutive days at the dose of 5 mg/kg (twice daily) significantly lowered the concentration of H_2_O_2_ in the colon of TNBS-treated mice indicating a substantial reduction in the oxygen free radicals (Fig. 3c).

## Discussion

In this study, we characterized the chemical composition of the EP pomace polyphenol extract, for which the source material is a residue that remains after extraction of oil from EP seeds. We also examined the anti-inflammatory activity of the EP pomace polyphenol extract in a well-established mouse model of TNBS-induced colitis. Moreover, we attempted to investigate the potential mechanism of the anti-inflammatory effect.

Using UPLC-Q-TOF-MS analysis, we found that the major compounds present in the EP pomace polyphenol extract were pentagalloyl glucose, ellagic acid, catechin, and gallic acid, with several accompanying polyphenols at lower concentrations. Our results are in line with studies by Kiss et al. (2011); Kiss and Naruszewicz 2012, who showed that pentagalloyl glucose, catechin, and gallic acid were the main compounds in the EP seed extracts. Studies on polyphenols present in the EP extracts showed that these compounds are potent antioxidants; they may also affect the innate immunity by interaction with several pro-inflammatory pathways in the cell (Montserrat-de la Paz et al. 2012; Wettasinghe et al. 2002).

Encouraged by this first evidence that the polyphenol extract from EP pomace contains high concentrations of potentially beneficial anti-inflammatory compounds, we proceeded by establishing its therapeutic activity in the mouse model of colitis. We used a hapten-induced mouse model of intestinal inflammation to evaluate the anti-inflammatory activity of EP pomace polyphenol extract after oral and i.c. administration. Further studies on the absorption of the EP pomace polyphenol extract constituents are necessary to delineate whether the effect is related to a local side of action only or involves a systemic component as well.

Our data demonstrate that EP pomace polyphenol extract alleviates experimental colitis in mice, and to the best of our knowledge, this is the first report on the anti-inflammatory action of the preparation. Based on our observations and previous reports, showing the improvement of UC in humans after treatment with EP seed oil, we may suggest that the application of EP preparations, in particular the polyphenol extract from EP pomace, may become a promising complementary therapy for patients suffering from IBD.

The anti-inflammatory and antioxidant properties of the EP pomace polyphenol extract may arise from the phenolic content and high sum of phenolic groups in total. This includes various phenolic acids, such as gallic and ellagic acid, pentagalloyl glucose and flavanols, catechin and epicatechin derivatives. This is in line with the study of Peschel et al. (2007), who reported that the EP pomace extract has a potent antioxidant activity, comparable to that of other commercially available plant antioxidant materials, such as green tea or grape seed. Moreover, Choiu et al. (2012) demonstrated that the derivatives of catechin gallate, which we detected in the EP preparation, potently suppress dextran sulfate sodium-induced colitis and colon tumorigenesis in mice (Choiu et al. 2012). In contrast to Peschel et al. (2007), who only used in vitro assays, we extended this observation to the in vivo conditions and demonstrated for the first time that the EP pomace polyphenol extract displays anti-inflammatory and antioxidant action in the mouse GI tract.

The oxidative stress has been implicated in IBD pathogenesis. There are several antioxidative mechanisms in the GI tract, which include enzymes, such as catalase and superoxide dismutase, as well as nonenzymatic scavengers like glutathione, flavonoids, and/or polyphenols. During intestinal inflammation, concentration of H_2_O_2_ in the colonic tissue is increased. In our study, the level of H_2_O_2_ was increased in TNBS-treated animals when compared to the control group, and the administration of EP pomace polyphenol extract reversed this change. Our data suggest that EP pomace polyphenol extract may have protective properties against free oxygen radicals, possibly via the upregulation of cellular antioxidant enzymes.

Surprisingly, we observed only a minor change in IL-1β and no change in TNF-α levels upon treatment with EP pomace polyphenol extract, what may suggest the lack of influence of the preparation on the immune system. This may result from low concentration of unsaturated fatty acids, such as cis-linoleic acid, found in the preparation, which are known to act directly on immune cells to reduce inflammation (Bayles and Usatine 2009). Our preparation was nearly completely defatted during the process of pomace production and subsequent extractions. However, our observation needs to be investigated further. It is well established that the level of cytokines, such as TNF-α, is elevated during experimental colitis (Ouyang et al. 2012; Xiong et al. 2013), while there were no changes in TNF-α level observed in this study. We may hypothesize that this was specific for our laboratory/animal house conditions or strain of animals used in this particular set of assays.

One of the potential targets for the EP pomace polyphenol extract may be cyclooxygenase-2 (COX-2), which plays a significant role in inflammation. Although the therapeutic effectiveness of COX-2 inhibitors in experimental colitis is controversial, some papers support their beneficial effects when administered specifically to the colon (Lee et al. 2014). Docking studies showed that gallic acid binds in the active site of COX-2 at the nonsteroidal anti-inflammatory drug-binding site (Verma et al. 2013), where its carboxylate moiety interacts with Arg120 and Glu524. On the other hand, it is well known that various pro-inflammatory mediators are upregulated in the colon epithelium of IBD patients. One of the key players in the exacerbation of inflammation in the colon is the nuclear transcription factor-kappaB (NF-κB), whose activation upregulates expression of many genes involved in immunity and inflammation (Verma et al. 2013). In a noninflamed tissue, the activity of NF-κB is suppressed by a specific inhibitor, IκB. It has been demonstrated that treatment with gallic acid produced a dose-dependent increase in IκB in vitro, thus inhibiting the activity of NF-κB and the pro-inflammatory cascade (Ho et al. 2010). Well-established inhibitors of NF-κB, such as salicylates (e.g., 5-ASA) and curcumin alleviated symptoms of IBD in both animal models and humans (Suskind et al. 2013). This may also be the case for the EP pomace polyphenol extract.

The results of our study encourage further investigations on the polyphenol extract from the EP pomace and possible translation of our findings into clinical conditions for the benefit of patients with IBD. Therefore, safety, stability, and additional in vivo activity tests for the polyphenol extract from the EP pomace, especially in combination with other natural antioxidants, are solicited.

## Conclusion

EP pomace polyphenol extract, derived from the waste material which remains after EP oil extraction, has a potent anti-inflammatory action in the GI tract. Moreover, we showed that both oral and i.c. administrations alleviate intestinal inflammation, thus making the EP pomace an attractive novel supplement for future anti-inflammatory treatment in IBD patients.
